# The Importance Of Multi-site Intra-operative Tissue Sampling In The Diagnosis Of Hip And Knee Periprosthetic Joint Infection - Results From A Single Centre Study

**DOI:** 10.7150/jbji.39499

**Published:** 2020-05-18

**Authors:** Lucy C. Walker, Nick D. Clement, Ian Wilson, Munawar Hashmi, Julie Samuel, David J. Deehan

**Affiliations:** 1 Freeman Hospital, Newcastle-upon-Tyne Hospitals NHS Foundation Trust, Freeman Road, Newcastle-upon-Tyne, NE7 7DN, UK; 2 Institute of Genetic Medicine, Newcastle University, International Centre for Life, Newcastle-upon-Tyne, NE7 7DN, UK

**Keywords:** Periprosthetic joint infection, intra-operative samples, culture, predictive model

## Abstract

**Introduction**: The primary aim of this study was to determine whether the tissue type and anatomical location of intra-operative samples influences the accuracy of culture in the diagnosis of periprosthetic joint infection (PJI). The secondary aim was to create a predictive model of PJI using other known patient variables.

**Methods**: A retrospective cohort of 3460 intra-operative samples from 887 patients was identified. The data was then analysed to compare intra-operative culture results (positive or negative) to the chosen gold standard of clinical diagnosis made by the treating team (infected or non-infected prosthetic joint). The intra-operative samples were grouped according to their labelling at the time of collection.

**Results**: No single tissue type or anatomical location had both high sensitivity and specificity. The highest specificity for an anatomical location was hip bursa with 100%, for tissue type it was synovium with 93%. Sensitivity was highest in the anatomical locations for hip capsule (68%) and in the tissue types for pus (83%). Data analysis was performed to create a model for PJI and identified pre-operative predictors of PJI (increased white cell count, knee joint and non-revision surgery) which when used in combination with intra-operative culture results increased the sensitivity.

**Conclusion**: Sample type and anatomical location influenced the reliability of the diagnosis of PJI however, no single sample type had higher diagnostic accuracy than samples combined thereby highlighting the necessity of obtaining multiple intra-operative samples in the diagnosis of PJI. The variation in predictive values of tissue types as well as improvement in sensitivity when combined with patient factors indicates that types of intra-operative sampling and the overall diagnostic pathway should vary depending on the individual case.

## Introduction

Periprosthetic joint infection (PJI) is the second most common complication of joint arthroplasty 1, with a reported occurrence of 0.94% of primary total hip and knee arthroplasties in the UK 2. Culture results are considered extremely useful as they not only provide a diagnosis but also identify the specific pathogens and sensitivity profiles 3-5. An incorrect diagnosis of PJI may lead to an unnecessary surgical procedure 6 whereas not recognising PJI will result in early implant failure with an untreated infection 7.

In order to maximise the accuracy of periprosthetic tissue culture, research has focused on numerous factors including types of samples 8-10, 11-13, culture methods 5, 14, 15 and methods of sampling 16. The surgeon is currently advised to use a different set of instruments to minimise contamination with suspected infection and it is recommended that a minimum of three samples are taken during surgery 3.

Regarding the specific tissue type or anatomical location of the samples, the evidence base is lacking and associated guidance is vague. Tissue samples have been shown to have superior sensitivity and specificity compared to deep intra-operative swab samples in cases where tissue samples were selected in the areas that appeared most inflamed and infected 17. Sampling of joint fluid through aspiration has variable results with a wide range of reported sensitivities (50-93%) and specificities (82-97%) 18. It is acknowledged that superficial swabs are poor quality samples with low sensitivity and specificity 8. Superficial wound fluid samples have also been shown to have poor accuracy in the diagnosis of PJI. Evidence suggests that in order to maximise the sensitivity and specificity of intra-operative specimen cultures at least five or six specimens should be obtained during surgery 19.

It has also been reported that adjunct investigations and patient factors can be used in combination to determine an individual's risk of having a PJI 20. Similarly, a logistic regression model has also been designed to predict the likelihood of surgical debridement successfully treating PJI 21. These predictive models can be used to support clinical decision making and as well as during patient counselling.

The primary aim of the current study was to examine the impact of anatomical site and tissue sample type in the diagnosis of hip and knee PJI. The secondary aim was to create a prediction model of PJI incorporating patient factors along with serum inflammatory markers and microbiology results, which have previously been used to predict the risk of PJI 22. The null hypothesis was that the sensitivity and specificity of intra-operative samples are not affected by sample type or anatomical location.

## Materials and Methods

### Method Design

This was a retrospective cohort study investigating the effect of tissue type and anatomical location of intra-operative samples on the accuracy in the diagnosis of PJI. The standard for comparison was the clinical diagnosis made by the treating surgical team of an infected or non-infected prosthetic joint. A prediction model for PJI was then created using pre-operative patient factors in isolation and also in combination with intra-operative cultures. This study was conducted in collaboration with orthopaedic surgeons, microbiologists and diagnostic laboratory services at a tertiary centre.

### Identification of microbiology samples

A consecutive cohort of all microbiology intra-operative periprosthetic samples taken from hips or knees between January 2010 and December 2016 was identified. The patient cohort was identified from the Laboratory Information Management System (LIMS) by using search tool Cognos 23 to identify all culture samples codes logged from patients who had been admitted to one of the centre's two orthopaedic wards at the time the sample was taken. For the seven year search period 4451 individual sample codes were identified (2010 n=510, 2011 n=551, 2012 n=585, 2013 n=713, 2014 n=770, 2015 n=789, 2016 n=533). The data search was performed by a laboratory technician and then crosschecked by an orthopaedic research fellow. Each sample code also had a corresponding sample label and final culture result with an isolated organism if positive. These codes were then cross referenced using a separate microbiology laboratory system, Laboratory Medicine Results, to identify the unique patient numbers linked to each sample. The data was then sorted according to unique patient identification number.

### Patient Cohort

**Figure 1:**
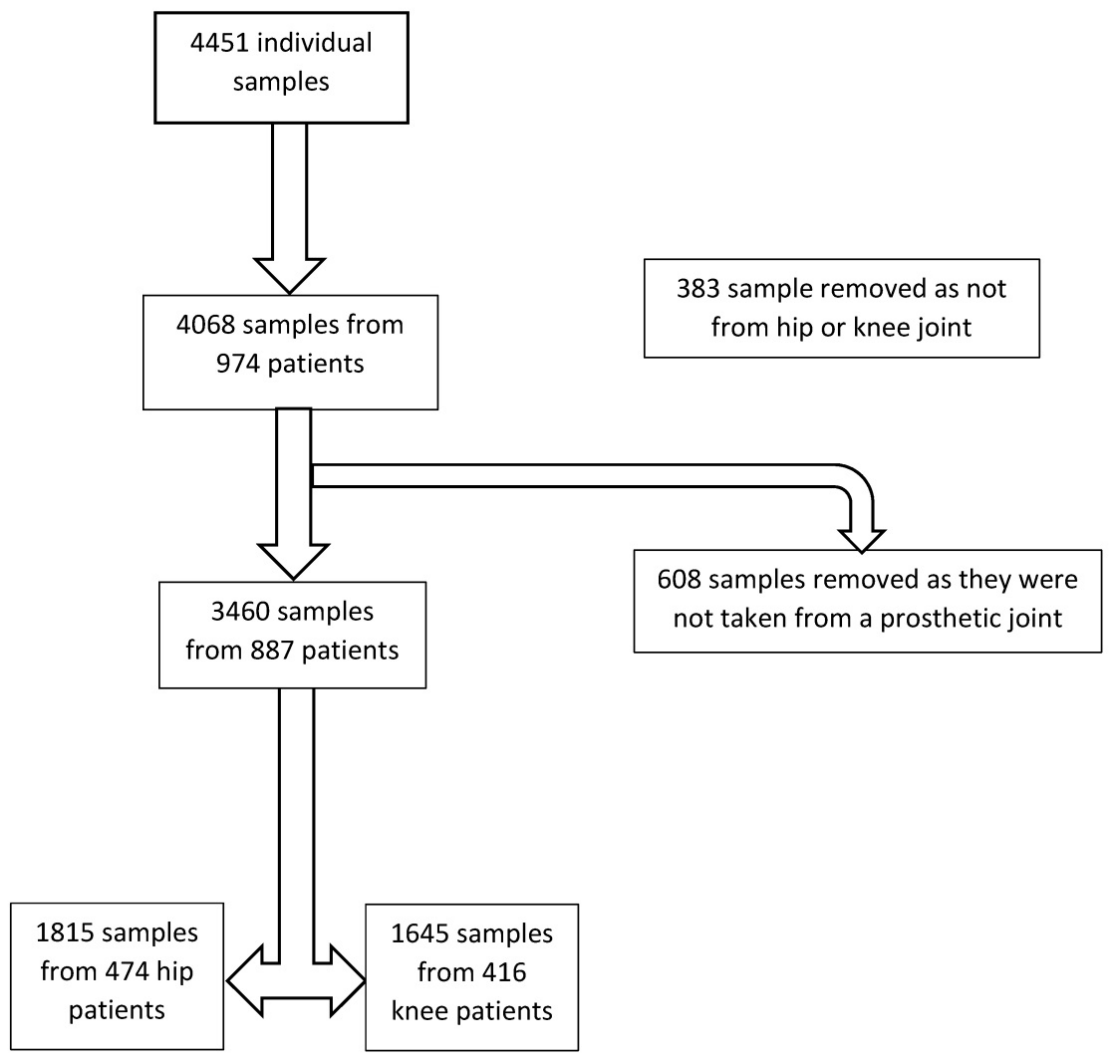
Flow chart showing development of final patient cohort from initial data set

Figure 1 illustrates how the final cohort was formed. From the initial cohort of 4451 samples, 383 samples were removed after initial review as the sample label indicated the sample was not taken from either a hip or knee, and a further 608 samples were removed as they were not taken from a prosthetic hip or knee joint (i.e. were taken from a native joint). This left a remaining cohort of 3460 sample codes from 887 patients.

These 887 patient numbers were then entered into the centre's electronic records system, which contains information on laboratory results, radiological investigations, discharge letters and clinic letters. Combined with the microbiology sample information from LIMS each individual patient had data collected for: date of birth, sex, past medical history, orthopaedic history, pre-operative white blood cell (WBC) count, C-reactive protein (CRP) and erythrocyte sedimentation rate (ESR), type of surgery performed at time of sampling, type of sample and request information (i.e. site/tissue type/method of collection), culture results, organisms identified and sensitivities (if culture positive: all specimens that had grown a micro-organism and had sensitivities available were considered positive irrespective of any query regarding contamination), clinical diagnosis of PJI versus aseptic joint and subsequent management plans. Due to the retrospective design of the study data was not collected regarding when the specimens were collected in relation to the stage of the procedure and antibiotic administration, nor was information available regarding the surgeon's reasoning for selecting a particular sample type, for example whether the area looked clinically infected.

The final cohort of 3460 samples taken from 887 patients with 1076 patient-operation pairs provided the material to test the null hypothesis that sample tissue type or anatomical location would not affect the diagnostic reliability for PJI, using the clinical diagnosis of PJI versus aseptic joint as the standard for comparison.

### Statistical analysis

Simple descriptive statistics were undertaken to calculate the sensitivity, specificity, positive predictive value (PPV) and negative predictive value (NPV) for intra-operative samples in diagnosing PJI. The sample culture test result (positive or negative) was compared against the clinical diagnosis of PJI (yes or no).

Statistical analysis was performed using Statistical Package for Social Sciences version 17.0 (SPSS Inc., Chicago, IL, USA). A Student's t-test and ANOVA were used to compare continuous variables between groups. Dichotomous variables were assessed using a Chi square test. A *p* value of <0.05 was defined as significant.

A logistic regression model 24 was fitted using the glm (generalised linear model) function in R 25 to clinical diagnosis (gold standard) using the covariates: age, WBC count, revision, joint and sample tissue type, with and without the intra-operative sample culture result. Model selection was performed using the Bayesian Information Criterion (BIC) 26, and the best models with and without the culture results for prediction of PJI were compared. The success of predictions can be visualised using a Receiver Operating Characteristic (ROC) curve for the model which shows the relationship between false positive predictions and false negative predictions.

### Ethical statement

The authors conducted a retrospective service evaluation, as such there was no additional patient contact and no requirement for formal ethical approval. The project was registered with the institutions audit department (registration number 7851) and was conducted in accordance with the Declaration of Helsinki and the guidelines for good clinical practice.

## Results

**Figure 2:**
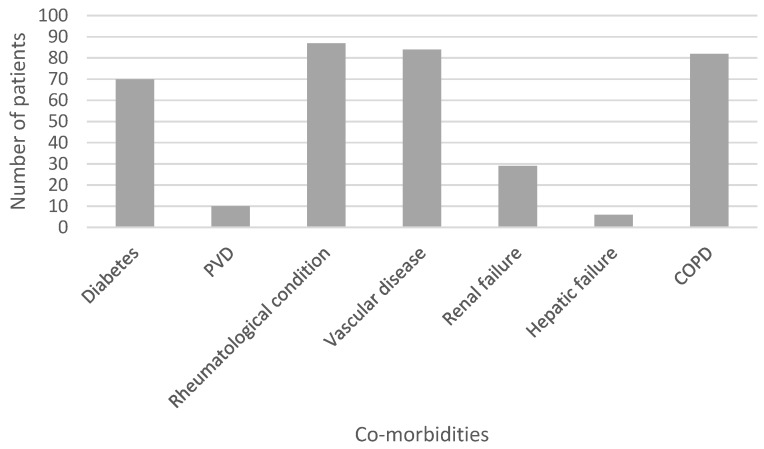
Bar chart showing the prevalence within the cohort of each co-morbidity associated with an increased risk of infection

The final cohort had 1815 samples taken during 557 operations from 474 patients undergoing hip surgery and 1645 samples taken during 519 operations from 416 patients undergoing knee surgery (three patients underwent both hip and knee surgery). The mean age was 68 years (range 24-94), with 493 female and 394 male. 314 patients had medical co-morbidities that could increase their risk of infection (diabetes, peripheral vascular disease, rheumatological conditions, vascular disease, renal failure, hepatic failure, chronic pulmonary disease 27). Figure 2 shows the rate of occurrence of these co-morbidities within the cohort.

Table 1 shows the patient demographics per operation type. Total hip arthroplasty (THA) revision was the most common hip surgery (N=164) and total knee arthroplasty (TKA) revision was the most common knee surgery (N=204). Table 2 shows comparison between the patient cohorts of hip and knee surgery. There was a statistically significant difference (p<0.05) between the two groups with regards to age, male:female ratio and proportion of patients with a clinical diagnosis of PJI. There was no statistically significant difference between the hip and knee surgery cohorts with regards to the proportion of patients with a medically increased risk of infection (p=0.1).

### Reliability of tissue type

**Figure 3:**
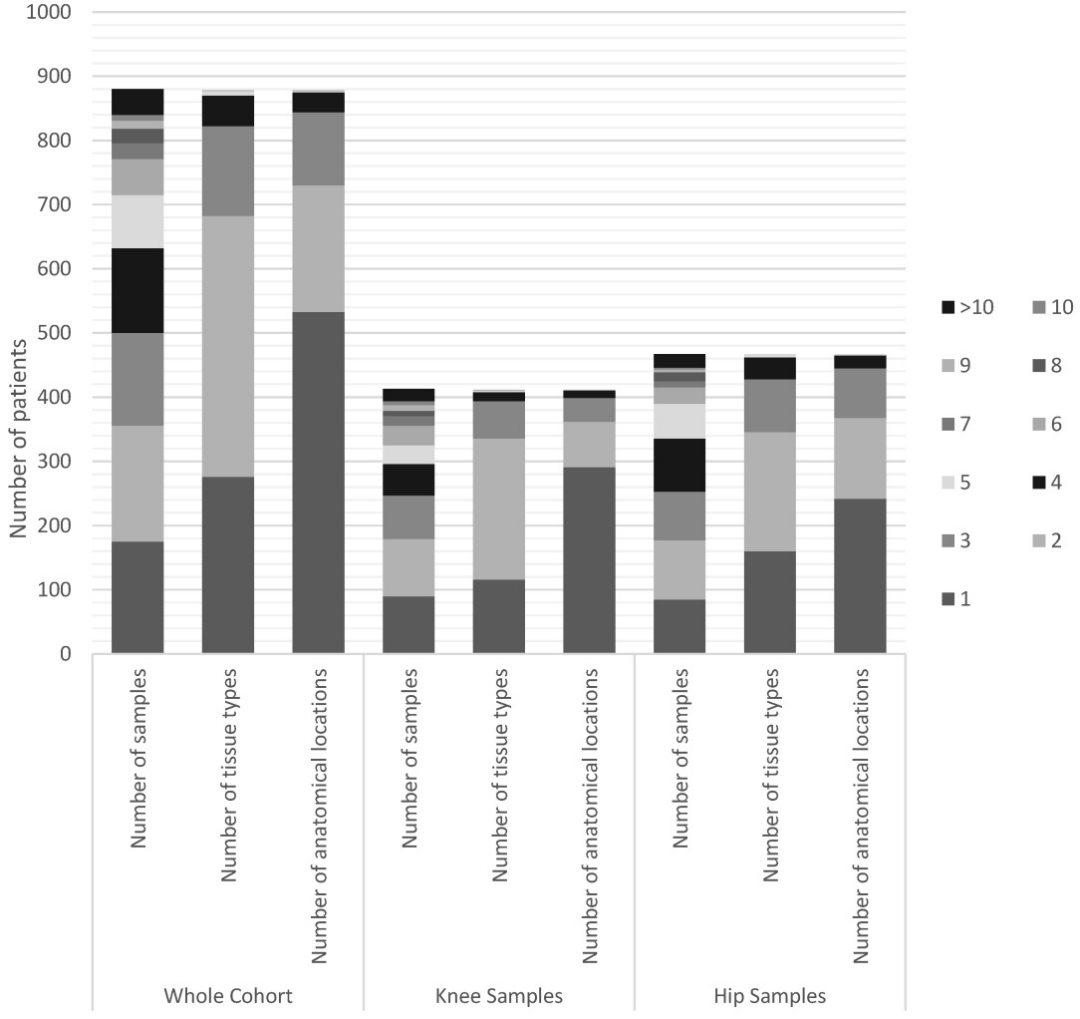
Distribution of the number and variability of samples taken intra-operatively across the cohort

There were 3460 samples taken from the 887 patient-operation pairs. There were 1645 samples taken from knees and 1815 from hips. 1411 hip samples and 1025 knee samples were taken from patients without a clinical diagnosis of PJI. 404 hip samples and 620 knee samples were taken from patients with a clinical diagnosis of PJI. Table 3 shows the mean number of samples taken within the cohort. Figure 3 shows the variability in the number of samples taken within the cohort.

Table 4 shows the sensitivities, specificities, PPVs and NPVs for samples as per tissue type. Pus had the highest sensitivity (83%) but the lowest specificity (67%). The highest specificity was for synovium (93%). Table 4 shows the diagnostic accuracy of samples as per the anatomical location. Hip capsule had the highest sensitivity (68%) and there was 100% specificity for hip bursa. Knee femur and tibia also had high specificities (90% and 89% respectively) but low sensitivities (32% and 34% respectively).

### Predictive model

The cohort as a whole was then analysed to create a model for PJI using pre-operative factors: increased medical risk of infection, age, type of joint, pre-operative inflammatory markers (WBC count, CRP and ESR) and whether the operation was a revision surgery. The best fitting model included the covariates: WBC count, whether the operation was a revision, and the sample tissue type. Age did not improve the fit of the model. Revisions (single, first or second stage) decreased the probability of infection.

Adding culture results to the prediction model improved the fit considerably, and again comparison of the Bayesian information criterion (BIC) for the models gave a best model with WBC count, revision and sample tissue type and additionally, the culture result. All of these co-variants were significant with p<0.0001.

**Figure 4:**
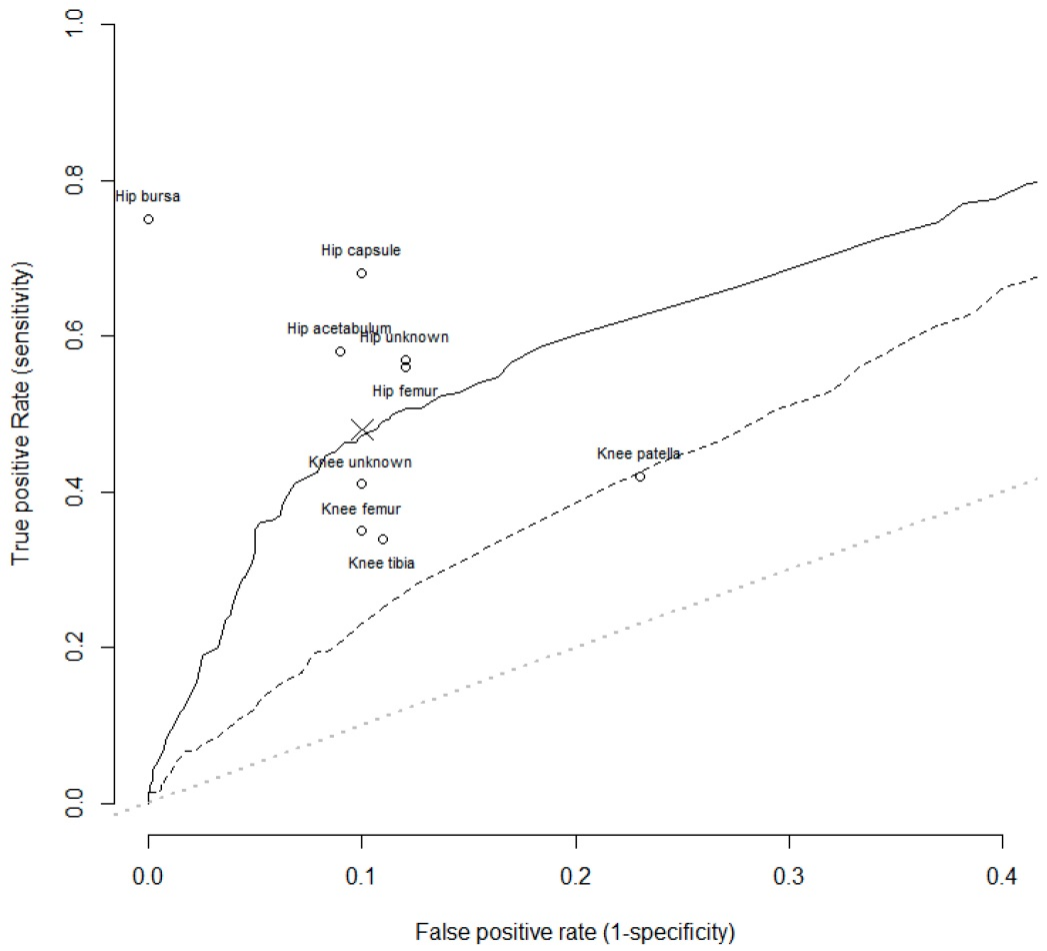
ROC curve analysis of PJI predictor model without culture information (dashed line, middle line) and using culture information (solid line). The lower dotted line indicates a test with no prognostic power. Circles are from the raw data for tissue types, the cross gives the overall sensitivity and specificity.

Figure 4 shows the ROC curve for the PJI model. Using the results for tissue alone the false positive rate clustered around 10%. To create a model with a superior false positive rate i.e. 5%, the true positive rates of the model then decreased. Therefore, it appeared that no model is possible that produces improved false positive rates without compromising the true positive rates.

## Discussion

The key finding of this investigation is that there is a variation in diagnostic accuracy between intra-operative samples with differing tissue types and anatomical location. However, no single sample type had diagnostic accuracy comparable to previously reported rates of sensitivity, specificity and predictive values of intra-operative culture samples considered collectively 17, thereby highlighting the necessity for sampling from multiple sites and tissue types in the diagnosis of PJI. It was also shown that by factoring in patients' pre-operative WBC count, type of surgery and culture result along with sample tissue type the best possible predictive model of PJI was created.

### Limitations

One limitation of this study is the retrospective design, so data could not be collected on the surgeons' decision making in selecting the samples types and sites, nor could the accuracy of the sample labelling be controlled. However, the dataset was collected independent of any specific hypothesis therefore excluding the potential for bias. Data was not collected regarding when the specimens were collected in relation to the stage of the procedure and antibiotic administration. Trampuz et al.^28^ demonstrated that any use of antibiotics in the two weeks before obtaining culture samples was associated with a lower yield of cultures from sonication samples obtained from hip and knee prostheses. This could therefore, have impacted upon the culture results of current studies samples. Within the study centre antibiotics are routinely held until after culture samples are taken, although data regarding compliance within the protocol or pre-operative antibiotic administration in the community were not available. The current study also included samples taken from patients during second stage revision surgeries who had previously had antibiotic-loaded cement spacers in situ and systemic antibiotics. It is possible persistent antibiotic elution may yield false negative culture results 29. However, it has also been shown that new infective organisms can be identified between explantation and re-implantation 30, 31 so the authors felt it important that these samples were included in the analysis as a new PJI pathogen can be diagnosed at the second stage surgery.

It could also be argued that the choice of the documented clinical diagnosis of PJI as the gold standard for comparison is a limitation. The recorded diagnosis of PJI versus an aseptic joint was collected in the dataset based solely on the documentation of the treating clinical team, without reviewing or revising the basis of their diagnostic decision making. The majority of the current literature uses intra-operative culture results as one of the criterion for their gold standard but as that was the variable the current study was assessing a different standard was chosen. Furthermore, using culture results as a gold standard does have its limitations as false negative culture results have been reported with a frequency that ranges from 7-23% 32. The specific causative micro-organism can also have an influence on culture specificity, with virulent organisms such as *Staphylococcus aureus* growing easily 5 whereas atypical organisms do not grown on routine media and organisms such as *Propionibacterium acnes* need one to two weeks for isolation 33-36. There are accepted diagnostic criteria available that could have been selected as a gold standard, such as the Musculoskeletal Infection Society's (MSIS) Workgroup criteria 5. However, this was not used in the current study as the majority of patients did not have all investigations completed to use this criteria. Furthermore, Honkanen et al.^37^ have suggested that true PJI cases could be missed by the MSIS criteria as a result of exclusion of clinical decision making.

The majority of samples analysed in the current study had been taken from patient without a clinical diagnosis of PJI. The majority of cases also had one to three samples taken, rather than the recommended five or six samples 19. This raises the question of the clinical reasoning behind taking samples if cases were clearly of a non-infected nature and the amount of resources being spent on these tests. It has previously been reported that up to £23 900 000 was spent within the NHS on unnecessary respiratory microbiological testing 38 and this may reflect a similar issue within orthopaedics. 129 samples were taken from primary hip replacements and 87 samples were taken from primary knee arthroplasty which would be presumed to be non-infected however, there were five cases of PJI cases in primary total hip arthroplasties although unfortunately further details regarding these cases were not available.

### Diagnostic accuracy of samples per tissue type and location

The current study found fluid samples to have a low sensitivity but high specificity, which has previously been reported by Gallo et al.^39^ Synovial tissue samples in the current study also had low sensitivity which is comparable to the findings of Cross et al.^40^ and questions the utility of pre-operative synovial biopsies. Collectively intra-operative cultures in this study had much lower sensitivity than the results reported by Fink et al.^16^ for tissue biopsy results, however, they were assessing pre-operative percutaneous tissue samples rather than those taken intra-operatively.

The tissue type and anatomical location of samples has been shown by the current study to have varying predictive values for PJI. For tissue type, pus had the highest PPV although there was a small sample size for this group. Capsule had the most superior NPV. For hips, bursa samples had high PPV and NPV. It is recommended that tests with high NPV be used to screen for infection and those with high PPV be used to confirm a diagnosis of PJI 41, and therefore the findings of the current study could guide treating surgeons on which sample types should be taken depending on their level of clinical suspicion of PJI. The variation in diagnostic accuracy across all sample types also highlights the need for multi-site sampling in the diagnosis of PJI. This supports the MSIS criteria that recommends a minimum of three intra-operative samples are taken 3.

### Predictive model for PJI

Using co-variates to create a predictive model for PJI showed pre-operative WBC count, type of surgery (revision versus non-revision) and sample tissue joint type (knee or hip) to be the best fit. Patients with non-revision (type of surgery) and WBC count >10 x 109 cells per litre and tissue from knee joints were found to be at increased risk of a PJI diagnosis. The finding that knee prostheses have an increased risk of PJI correlates with Pulido et al.^42^ who also reported a higher incidence of PJI in knee replacements than for hip replacements. Serum WBC count rises in response to infection, therefore would be expected to be raised in PJI. However, its use as a single diagnostic marker of PJI has been discouraged due to its poor sensitivity and specificity 43, 44. Revision surgery decreasing the risk of a PJI diagnosis could be explained by the routine surgical practice of taking intra-operative samples for all revision surgeries, even those with a very low clinical suspicion of infection. Therefore, if only an isolated sample was culture positive but there was no clinical suggestion of infection then it can still be considered an aseptic joint. Whereas, for primary surgery samples are not routinely taken, therefore in the cases that do have cultures sent it may be due to pre-existing clinical suspicion of infection. When using these factors in combination with intra-operative culture results for predicting PJI the sensitivity improved but the specificity decreased. Therefore, in order to increase the sensitivity of a PJI diagnosis it is useful to consider other diagnostic modalities, such as pre-operative inflammatory markers, patient and surgical factors.

The current study has demonstrated a variation in sensitivity, specificity and predictive values according to intra-operative sample type. Due to the variation in predictive values, the operating surgeons may wish to alter which intra-operative sample types are selected depending on their level of clinical suspicion, as well as considering pre-operative patient and joint variants in the diagnostic process.

## Conclusion

In conclusion, this study analysed intra-operative samples collected irrespective of type and presence of infection and irrespective of the surgeon's reasoning for selecting a particular sample type. Whilst this did demonstrated variation in diagnostic accuracy depending on the tissue type and anatomical location of intra-operative culture samples taken to diagnose PJI, no individual tissue or anatomical type had superior accuracy compared to all samples used collectively. However, the diagnostic reliability was increased when pre-operative inflammatory markers and surgical factors (type or joint and primary or revision arthroplasty) were incorporated into a predictive model for PJI.

## Author Contributions

LCW performed the investigation, data curation and writing of the original draft manuscript and re-editing. NDC reviewed and edited the manuscript. IW performed the formal data analysis. MH supervised the project as well as reviewing the manuscript. JS helped devise the metholodology and data curation by allowing access to the necessary software. DJD devised the concept of the project and provided supervision throughout as well as reviewing and editing the manuscript.

## Figures and Tables

**Table 1:** The final patient cohort divided per operation

	Patient demographics			
	Average age (years)	Sex M:F	% of patients with medical co-morbidities	% with clinical diagnosis of PJI
**HIP SURGERY**				
Primary total hip arthroplasty (THA) (n = 75)	65.3	33:42	45.3%	6.7%
THA revision (single stage) (n = 164)	70.0	78:86	28.7%	4.3%
THA 1^st^ stage revision (n = 23)	65.9	16:7	39.1%	100%
THA 2^nd^ stage revision (n = 21)	66.7	12:9	19.0%	9.5%
THA washout/debridement (n = 31)	71.2	16:15	32.3%	71.0%
THA aspiration (n = 9)	66.6	3:6	22.2%	44.4%
Dynamic hip screw (DHS) converted to THA (n = 22)	74.3	2:20	63.6%	0%
Acetabulum revision (n = 138)	68.5	45:93	26.1%	2.2%
Stem revision (n = 11)	71.1	6:5	45%	9.1%
Hemiarthroplasty revised to THA (n = 20)	77.1	2:18	45%	5%
Hemiarthroplasty (n = 3)	82.3	1:2	33.3%	66.7%
Resurfacing revised to THA (n = 23)	52.6	9:14	30.4%	0%
Statistical difference - *ANOVA **Chi squared test	0*	0.0003**	0.02184**	0**
All hip surgery (n = 557)	68.6	229:328	33.0%	14.5%

**KNEE SURGERY**				
Primary total knee arthroplasty(TKA) (n = 51)	66.8	27:24	45.1%	0%
TKA revision (single stage) (n = 204)	69.0	108:96	39.2%	7.8%
TKA 1^st^ stage revision (n = 66)	65.9	44:22	41.0%	100%
TKA 2^nd^ stage revision (n = 39)	64.8	24:15	28.2%	28.2%
TKA washout/debridement (n = 55)	62.0	29:26	43.6%	61.8%
TKA aspiration (n = 6)	63.4	5:1	66.6%	66.6%
TKA patellar resurfacing (n = 60)	66.5	19:41	31.7%	1.7%
Arthrodesis (n = 7)	63.4	5:2	38.6%	38.6%
Arthrodesis revised to TKA (n = 2)	60.6	1:1	0%	0%
Distal femoral replacement (n = 2)	80.8	2:0	0%	0%
Femoral component revision (n = 2)	60.0	2:0	0%	0%
Patellofemoral replacement (n = 2)	59.3	0:2	0%	0%
PF replacement revised to TKA (n = 12)	61.1	2:10	33.3%	0%
Tibial revision (n = 2)	69.8	0:2	0%	0%
Uni revised to TKA (n = 9)	67.5	4:5	22.2%	0%
Statistical difference - ANOVA* Chi squared test**	0.00756*	0.001**	0.47336**	0**
All knee surgery (n = 519)	66.6	272:247	37.8%	25.8%
TOTAL ALL SURGERIES (n = 1076)	68.2	501:575	35.3%	20.0%

**Table 2:** Patient demographics of hip and knee surgery cohorts

	Hip surgery (n = 557)	Knee surgery (n = 519)	Statistical difference (Students T-test*, Chi-squared test**)
**Average age (years)**	68.6	66.6	p=0.007*
**Male:female ratio**	229:328	272:247	p=0.0002**
**% with medical co-morbidities increasing risk of infection**	33.0%	37.8%	p=0.104718**
**% with diagnosis of PJI**	14.5% (N=81)	25.8% (N=132)	p<0.001*

**Table 3:** Number of intra-operative samples taken from the study cohort

	Total cohort	Knee cohort	Hip cohort
Mean number of samples (range)	3.93 (1-50)	3.99 (1-50)	3.88 (1-25)
Mean number of tissue types sampled (range)	1.98 (1-6)	1.95 (1-6)	2.01 (1-5)
Mean number of anatomical locations sampled (range)	1.60 (1-6)	1.45 (1-6)	1.75 (1-5)

**Table 4:** Diagnostic accuracy divided by tissue type and anatomical location

	Number of samples (number of patients)	Sensitivity	Specificity	PPV	NPV
**Tissue type**					
**Bone**	90 (68)	45% (30-61)	85% (71-94)	74%	62%
**Capsule**	185 (113)	61% (41-78)	90% (85-95)	53%	93%
**Fluid**	928 (660)	39% (33-46)	92% (90-94)	64%	82%
**Membrane**	207 (140)	43% (26-61)	86% (80-91)	38%	88%
**Pus**	15 (13)	83% (52-98)	67% (9-99)	91%	50%
**Synovium**	131 (87)	41% (25-59)	93% (86-97)	67%	82%
**Tissue**	1886 (654)	50% (46-54)	88% (86-90)	68%	78%
**Anatomical location**					
**All hip**	1815 (473)	58% (53-63)	89% (88-91)	61%	88%
**Hip acetabulum**	337 (188)	58% (44-71)	91% (88-94)	58%	91%
**Hip bursa**	13 (12)	75% (19-99)	100% (66-100)	100%	90%
**Hip capsule**	173 (101)	68% (48-84)	90% (84-95)	58%	94%
**Hip femur**	167 (113)	56% (41-71)	88% (81-93)	66%	83%
**All knee**	1645 (417)	41% (37-45)	90% (88-92)	71%	71%
**Knee femur**	120 (76)	35% (22-50)	90% (80-96)	72%	65%
**Knee patella**	34 (26)	42% (15-72)	77% (55-92)	50%	71%
**Knee tibia**	92 (72)	34% (19-52)	89% (78-96)	67%	69%

Uncertainty of estimates in brackets

## Abbreviations

PJI: Periprosthetic joint infection
LIMS: Laboratory information management system
WBC: White blood cell
CRP: C-reactive protein
ESR: Erythrocyte sedimentation rate
PPV: Positive predictive value
NPV: Negative predictive value
SPSS: Statistical package for social sciences
BIC: Bayesian information criterion
ROC: Receiver operating characteristic
THA: Total hip arthroplasty
TKA: Total knee arthroplasty
MSIS: Musculoskeletal infection society
